# Supplementary material for: A fault-intrusion-tolerant system and deadline-aware algorithm for scheduling scientific workflow in the cloud
Source: PeerJ Comput Sci. 2021 Nov 2;7:e747. doi: 10.7717/peerj-cs.747 (PMC8576554; doi:10.7717/peerj-cs.747)
Supplement: Supplemental Information 6 [file peerj-cs-07-747-s006.zip › Myproject1.1/docs/org/cloudbus/cloudsim/class-use/DatacenterCharacteristics.html]

Uses of Class org.cloudbus.cloudsim.DatacenterCharacteristics


JavaScript is disabled on your browser.


- Overview
- Package
- Class
- Use
- Tree
- Deprecated
- Index
- Help

- Prev
- Next

- Frames
- No Frames

- All Classes

## Uses of Class org.cloudbus.cloudsim.DatacenterCharacteristics

- Packages that use DatacenterCharacteristics

  | Package | Description |
  | --- | --- |
  |  |  |
  | --- | --- |
  | org.cloudbus.cloudsim |  |
  | org.cloudbus.cloudsim.network.datacenter |  |
  | org.cloudbus.cloudsim.power |  |
  | org.workflowsim |  |
- - ### Uses of DatacenterCharacteristics in org.cloudbus.cloudsim

    Fields in org.cloudbus.cloudsim with type parameters of type DatacenterCharacteristics

    | Modifier and Type | Field and Description |
    | --- | --- |
    |  |  |
    | --- | --- |
    | `protected java.util.Map<java.lang.Integer,DatacenterCharacteristics>` | DatacenterBroker.`datacenterCharacteristicsList` The datacenter characteristics list. |

    Methods in org.cloudbus.cloudsim that return DatacenterCharacteristics

    | Modifier and Type | Method and Description |
    | --- | --- |
    |  |  |
    | --- | --- |
    | `protected DatacenterCharacteristics` | Datacenter.`getCharacteristics()` Gets the characteristics. |

    Methods in org.cloudbus.cloudsim that return types with arguments of type DatacenterCharacteristics

    | Modifier and Type | Method and Description |
    | --- | --- |
    |  |  |
    | --- | --- |
    | `protected java.util.Map<java.lang.Integer,DatacenterCharacteristics>` | DatacenterBroker.`getDatacenterCharacteristicsList()` Gets the datacenter characteristics list. |

    Methods in org.cloudbus.cloudsim with parameters of type DatacenterCharacteristics

    | Modifier and Type | Method and Description |
    | --- | --- |
    |  |  |
    | --- | --- |
    | `protected void` | Datacenter.`setCharacteristics(DatacenterCharacteristics characteristics)` Sets the characteristics. |

    Method parameters in org.cloudbus.cloudsim with type arguments of type DatacenterCharacteristics

    | Modifier and Type | Method and Description |
    | --- | --- |
    |  |  |
    | --- | --- |
    | `protected void` | DatacenterBroker.`setDatacenterCharacteristicsList(java.util.Map<java.lang.Integer,DatacenterCharacteristics> datacenterCharacteristicsList)` Sets the datacenter characteristics list. |

    Constructors in org.cloudbus.cloudsim with parameters of type DatacenterCharacteristics

    Constructor and Description

    `Datacenter(java.lang.String name,
    DatacenterCharacteristics characteristics,
    VmAllocationPolicy vmAllocationPolicy,
    java.util.List<Storage> storageList,
    double schedulingInterval)`

    Allocates a new PowerDatacenter object.
  - ### Uses of DatacenterCharacteristics in org.cloudbus.cloudsim.network.datacenter

    Methods in org.cloudbus.cloudsim.network.datacenter that return types with arguments of type DatacenterCharacteristics

    | Modifier and Type | Method and Description |
    | --- | --- |
    |  |  |
    | --- | --- |
    | `protected java.util.Map<java.lang.Integer,DatacenterCharacteristics>` | NetDatacenterBroker.`getDatacenterCharacteristicsList()` Gets the datacenter characteristics list. |

    Method parameters in org.cloudbus.cloudsim.network.datacenter with type arguments of type DatacenterCharacteristics

    | Modifier and Type | Method and Description |
    | --- | --- |
    |  |  |
    | --- | --- |
    | `protected void` | NetDatacenterBroker.`setDatacenterCharacteristicsList(java.util.Map<java.lang.Integer,DatacenterCharacteristics> datacenterCharacteristicsList)` Sets the datacenter characteristics list. |

    Constructors in org.cloudbus.cloudsim.network.datacenter with parameters of type DatacenterCharacteristics

    Constructor and Description

    `NetworkDatacenter(java.lang.String name,
    DatacenterCharacteristics characteristics,
    VmAllocationPolicy vmAllocationPolicy,
    java.util.List<Storage> storageList,
    double schedulingInterval)`

    Allocates a new NetworkDatacenter object.
  - ### Uses of DatacenterCharacteristics in org.cloudbus.cloudsim.power

    Constructors in org.cloudbus.cloudsim.power with parameters of type DatacenterCharacteristics

    Constructor and Description

    `PowerDatacenter(java.lang.String name,
    DatacenterCharacteristics characteristics,
    VmAllocationPolicy vmAllocationPolicy,
    java.util.List<Storage> storageList,
    double schedulingInterval)`

    Instantiates a new datacenter.

    `PowerDatacenterNonPowerAware(java.lang.String name,
    DatacenterCharacteristics characteristics,
    VmAllocationPolicy vmAllocationPolicy,
    java.util.List<Storage> storageList,
    double schedulingInterval)`

    Instantiates a new datacenter.
  - ### Uses of DatacenterCharacteristics in org.workflowsim

    Constructors in org.workflowsim with parameters of type DatacenterCharacteristics

    Constructor and Description

    `DatacenterExtended(java.lang.String name,
    DatacenterCharacteristics characteristics,
    VmAllocationPolicy vmAllocationPolicy,
    java.util.List<Storage> storageList,
    double schedulingInterval)`

- Overview
- Package
- Class
- Use
- Tree
- Deprecated
- Index
- Help

- Prev
- Next

- Frames
- No Frames

- All Classes
